# Glycemia in blood, brain and subcutaneous tissue measured by a continuous glucose monitoring system

**DOI:** 10.1186/cc9824

**Published:** 2011-03-11

**Authors:** M Zourek, Z Jankovec, P Hykova

**Affiliations:** 1 Faculty Hospital, Charles University, Plzen, Czech Republic

## Introduction

Continuous glucose monitoring system (CGMS) technology provides the opportunity to measure glycemia in different tissues [1]. The aim of our study was to determine the lag-time between blood, brain and adipose tissue during rapid glucose changes.

## Methods

Fifteen male hereditary hypertriglyceridemic rats underwent the experimental protocol. After intraperitoneal anesthesia, the internal jugular vein and carotid artery were catheterized. A CGMS sensor (Medtronic) was inserted into the brain by micromanipulators and to the abdominal subcutaneous tissue. At the beginning of the experiment (-120 minutes), basal glycemia was measured and calibration of the sensors was started. Thereafter, insulin infusion was started (50 mU/kg/minute) and 20% glucose at a variable rate of infusion. Blood glucose was measured every 5 minutes with manual correction of the glucose infusion rate to maintain the glycemia level of 6 mmol/l. At a time of -10 minutes, the calibration procedure was finished and actual glycemia was recorded to sensors. At a time of 0 minutes, a bolus of glucose 0.5 g/kg was administered; and at a time of 50 minutes, a bolus of insulin 5 IU/kg was administered. Moreover glucose and insulin infusion were stopped at this time. The experiment was finished at time 130 minutes and animals were euthanized.

## Results

After an intravenous glucose bolus of 0.5 g/kg, glycemia rose rapidly to 14 mmol/l in 5 minutes. On the contrary, the glucose content in the brain and subcutaneous tissue was increased in a slower manner, with a maximum in about 50 minutes (brain) and 60 minutes (subcutaneous tissue). Intravenous insulin bolus of 5 U/kg was followed by lowering blood glucose concentration to a minimum of 4.5 mmol/l. The brain and subcutaneous tissue glucose content decreased slowly to a minimum of 4.2 mmol/l (brain) and 5.5 mmol/l (subcutaneous tissue). The median glucose lag-time blood versus brain and blood versus subcutaneous tissue was 10 (10; 15) minutes and 15 (15; 25) minutes, respectively (*P *= 0.01).

## Conclusions

Contrary to a previous study, which showed no changes in glucose dynamics after a bolus of glucose between brain, adipose tissue and muscle, our data showed that glucose in the brain follows blood excursions during acute glycemic changes more closely compared with subcutaneous tissue [2].
